# Supplementary material for: Altered respiratory virome and serum cytokine profile associated with recurrent respiratory tract infections in children
Source: Nat Commun. 2019 May 23;10:2288. doi: 10.1038/s41467-019-10294-x (PMC6533328; doi:10.1038/s41467-019-10294-x)
Supplement: Supplementary file 1 — Supplementary Information [file 41467_2019_10294_MOESM1_ESM.pdf]

Altered respiratory virome and serum cytokine profile associated with recurrent respiratory tract infections in children

Yanpeng Li [1,2¶], Xuemin Fu[2¶], Jinmin Ma[3¶], Jianhui Zhang[1¶], Yihong Hu[2], Wei Dong[4], Zhenzhou Wan[5], Qiongfang Li[3], Yi-Qun Kuang[6], Ke Lan[2,#], Xia Jin[2], Jian-Hua Wang[2,*], Chiyu Zhang[1,2,*]

Supplementary Information

Supplementary tables

Supplementary Table 1. Characteristics of the ARTI children in this study.

	Total
Characteristics[#]	(n=4,407)
Median age(month, range)	50.0(1.0-157.0)
Gender(male/female)	2,563/1,844
Clinical symptoms[&]	(n=4,641)
Cough	76.0%
Fever	85.4%
Sore throat	43.6%
Running nose	64.4%
Expectoration	30.9%
White blood cell(10^9/L)	8.63(0.3-159.0)
Lymphocyte (%)	27.8(0.05-90.8)
Neutrophils (%)	60.2(0.29-97.0)
Hemoglobin(g/L)	123.0(1.0-709.0)
Platelet (10^9/L)	244.0(0.5-788.0)

Supplementary Table 2. Risk factors associated with multiple ARTIs (using first episode and Z-score transformation).

Predictive variables #	*P* value	Adjusted OR	95% CI	
			Lower	Upper
Z-score TIMP-1	<0.001	5.05	2.24	11.40
Z-score PDGF-BB	0.005	3.04	1.41	6.57
Z-score Eotaxin-1	0.105	2.06	0.86	4.92
Z-score ICAM-1	0.087	1.80	0.92	3.54
Age	0.034	1.56	1.03	2.37
Z-score TNFRII	0.100	0.57	0.29	1.11

#: First episode of each child with multiple ARTIs was used.

Supplementary Table 3. Risk factors associated with multiple ARTIs (using median and Log2 transformation).

Predictive variables #	*P* value	Adjusted OR	95% CI	
			Lower	Upper
Log2 TIMP-1	0.001	50.65	5.33	481.33
Log2 PDGF-BB	0.003	11.58	3.14	299.33
Log2 IL-17	0.124	0.75	0.52	1.08
Log2 IL-15	0.087	0.53	0.26	1.10
Log2 IL-6	0.018	0.42	0.20	0.86

#: The median of each child with multiple ARTIs was used.

Supplementary Table 4. Risk factors associated with multiple ARTIs (using median and Z-score transformation).

Predictive variables #	*P* value	Adjusted OR	95% CI	
			Lower	Upper
Z-score TIMP-1	<0.001	6.02	2.47	14.71
Z-score PDGF-BB	0.078	2.00	0.93	4.31
Z-score IL-6	0.119	0.58	0.30	1.15
Z-score IFN-b	0.038	0.51	0.27	0.93
Z-score IL-17	0.093	0.54	0.26	1.11
Z-score TNFRII	0.100	0.57	0.29	1.11

#: The median of each child with multiple ARTIs was used.

Supplementary Table 5. Comparison of the respiratory virome of ARTI between this study and previous studies.

	Publications	This study	Wang[1] (2016)	Graf[2] (2016)	Zhou[3] (2016)	Zoll[4] (2015)	Thorburn[5] (2015)	Wylie[6] (2014)	Lysholm[7] (2012)	Wylie[8] (2012)	Yang[9] (2011)	Willner[10] (2009)
	Subjects	ARTI Children	SARI/H Children	ARTI Children	ILI Children/Adults	ARTI/H Children/ Adults	URTI Adults	Healthy Adults	SLRTI Children/ Adults	Febrile/ Afebrile Children	LRTI Children	CF/not CF Adults
	Samples(n)	212 NPS	135/15 NPS	67 NPS	121 NPS	8/2 Sputum/NPA	89 NPS	706 Nose/Mouth	210 NPA	50/81 NPS/Plasma	16 NPA	5/5 Sputum
	Strategy[#]	Individual	Pooled	Individual	Individual	Individual	Individual	Individual	Pooled	Individual	Individual	Individual
Respiratory viruses	Anelloviridae	+	+	-	-	+	ND	+	+	+	-	-
	Adenoviridae	+	+	+	+	-	ND	+	+	+	+	+
	Coronaviridae	+	+	+	-	-	+	ND	+	+	-	ND
	Herpesvirinae	+	+	+	+	-	ND	+	-	+	+	+
	Papillomaviridae	+	+	-	-	-	ND	+	-	+	-	+
	Paramyxoviridae	+	+	+	+	-	+	ND	+	+	+	ND
	Parvoviridae	+	+	+	-	-	ND	+	+	+	+	+
	Picornaviridae	+	+	+	+	+	+	ND	+	+	+	ND
	Pneumoviridae	+	+	+	+	+	+	ND	+	+	+	ND
	Polyomaviridae	+	-	-	-	-	ND	+	+	+	-	ND
	Orthomyxoviridae	+	+	+	+	-	+	ND	+	+	+	ND
Phages	Microviridae	+	NA	NA	ND	ND	ND	ND	NA	ND	ND	+
	Myoviridae	+	NA	NA	ND	ND	ND	ND	NA	ND	ND	+
	Podoviridae	+	NA	NA	ND	ND	ND	ND	NA	ND	ND	+
	Siphoviridae	+	NA	NA	ND	ND	ND	ND	NA	ND	ND	+

Note: ARTIs: acute respiratory tract infections; SARI: severe acute respiratory tract infections; URTI: upper respiratory tract infections; ILI: influenza-like illness; SLRTI: severe lower respiratory tract infections; CF: cystic fibrosis; H: healthy; NPA: nasopharyngeal swab; NPA: nasopharyngeal aspirates; ND: not detected.
#: Sequencing libraries were constructed by either individual sample (this study) or pooled samples (e.g. using 15 samples mixture to make one library).

Supplementary Table 6. Detection of S. pneumoniae, H. influenza, and S. aureus by specific PCR assays$.

	The single-ARTI group (n=42)	The multiple-ARTIs group (n=45)	Total (n=87)	*P* value#
S. pneumoniae	17 (40.5%)	20 (44.4%)	37 (42.5%)	0.829
H. influenza	32 (76.2%)	34 (75.6%)	66 (75.9%)	1.000
S. aureus	25 (60.0%)	19 (42.2%)	44 (50.6%)	0.135

Comparision was performed using the Fisher's exact test.
$ The PCR methods were based on previous studies[11,12]. The primer details are listed in Supplementary Table 8. Briefly, PCR conditions for S. aureus are as follows: 94℃ for 3 min, followed by 30 cycles (94℃ for 30 s, 60℃ for 30 s and 72℃ for 30 s), followed by a final extension at 72°C for 5 min. PCR conditions for S. pneumoniae H. and influenza are as follows: 95℃ for 3 min, followed by 35 cycles (95℃ for 30 s, 50℃ for 60 s and 72℃ for 60 s), followed by a final extension at 72° C for 10 min.

Supplementary Table 7. Sequencing characteristics and distribution of reads based on the different taxonomy.

	Mean Reads/Percentage	IQR
Raw Data	37,646,191	31,851,706 – 37,425,259
Clean Data	37,037,694	31,298,294 – 36,956,720
Clean/Raw	98.36%	–
Human reads	2.86%	0.42% - 2.98%
Bacterial reads	21.27%	15.18% - 29.01%
Viral reads	0.23%	0.005% - 0.026%
Eukaryotic viruses	0.08%	0.001% - 0.011%
Bacteriophage	0.15%	0.002% - 0.010%

IQR: Interquartile range

Supplementary Table 8. Primers that used in the study

Virus	Primers & Probes	Sequences (5'-3')
HRV[13]	RV 445-sense	CCGGCCCCTGAATGYGGCTAA
	RV 1104-antisense	ACATRTTYTSNCCAAANAYDCCCAT
	RV 533-sense	ACCRACTACTTTGGGTGTCCGTG
	RV 1066-antisense	TCWGGHARYTTCCAMCACCANCC
HEV	EV-F	TCCTCCGGCCCCTGAAT
	EV-R	GAAACACGGACACCCAAAGTAGT
	EV-P	(TEX)AASTCTGYRGCGGAACC(MGB)
IFV	IFV(A)-F	GACCRATCCTGTCACCTCTGAC
	IFV(A)-R	AGGGCATTYTTGACAAAKCGTCTA
	IFV(A)-P	(FAM)TGCAGTCCTCGCTCACTGGGCACG(BQ1)
	IFV(B)-F	TCCTCAACTCACTCTTCGAGCG
	IFV(B)-R	CGGTGCTCTTGACCAAATTGG
	IFV(B)-P	(FAM)CCAATTCGAGCAGCTGAAACTGCGGT(BQ1)
RSV	RSV(A)-F	AATACAGCCAAATCTAACCAACTTTACA
	RSV(A)-R	GCCAAGGAAGCATGCAATAAA
	RSV(A)-P	TGCTATTGTGCACTAAAG
	RSV(B)-F	AATACAGCCAAATCTAACCAACTTTACA
	RSV(B)-R	GCCAAGGAAGCATGCAATAAA
	RSV(B)-P	(VIC)CACTATTCCTTACTAAAGATGTC(MGB)
S.pneumoniae[11]	ply-1	GACCCCAGCAATTCAATTCAAGTGT
	ply-2	TACGCACTAGTGGCAAATCG
H. influenza[11]	HiP6-F	ACTTTTGGCGGTTACTCTGT
	HiP6-R	TGTGCCTAATTTACCAGCAT
S. aureus[12]	F-Sa5	AAAGGTGTAGGTTGGAAAGTAGAAG
	R-Sa5	GTTACAGGCATTTTGTCTTTAGGTG

Supplementary figures

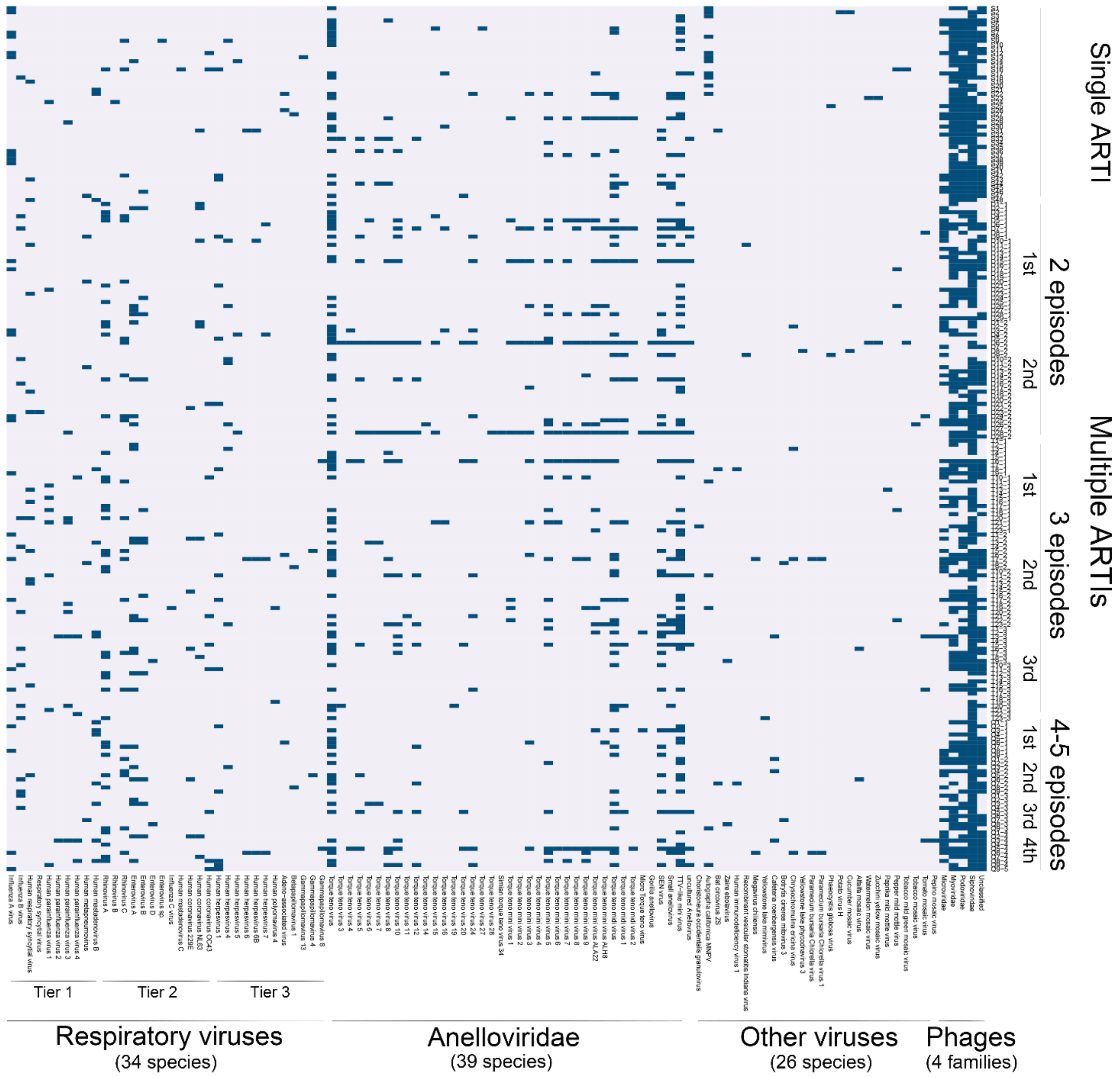

Supplementary Figure 1. The respiratory virome in ARTI children. The results were shown for each episode of children with single and multiple ARTIs. In total, 34 different common respiratory viruses, 39 anelloviruses and 4 main bacteriophage families were identified. The viruses detected in each sample was highlighted by a blue dash. According to the clinical characteristics, 34 respiratory viruses are grouped as tier 1: respiratory viruses with low proportions of subclinical infections, tier 2: respiratory viruses with high proportions of subclinical infections, tier 3: viruses rarely or not etiologically associated with ARTIs.

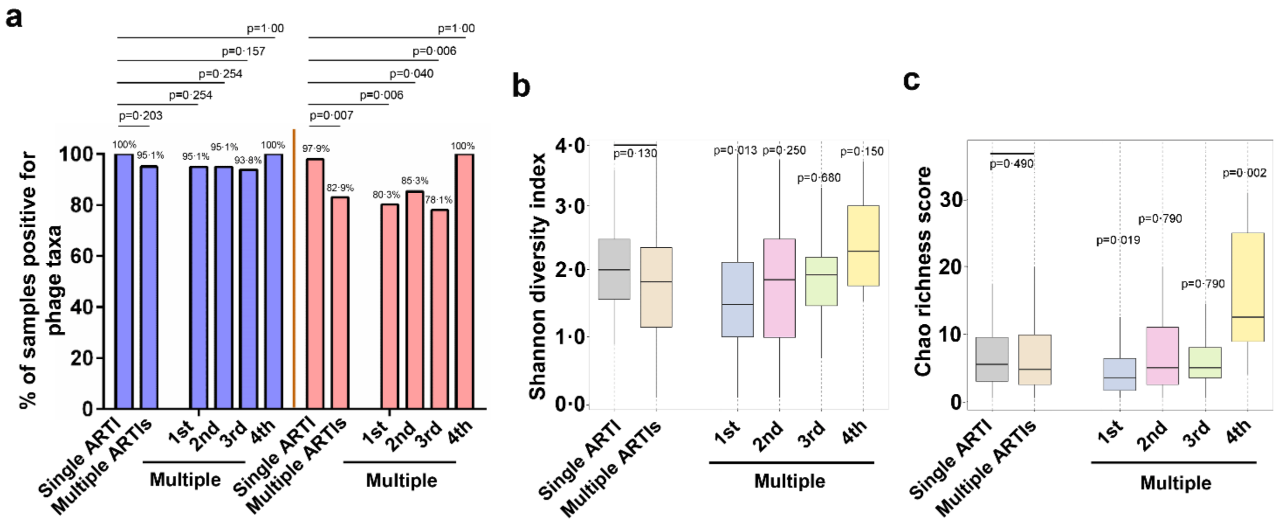

Supplementary Figure 2. Characteristics of bacteriophages among children with single or multiple ARTIs. (a)Detection (blue) and co-detection (red) rates of bacteriophages; (b) Alpha diversity (Shannon Diversity Index); (c) Richness (Chao Richness score). For other details, please see Figure 2.

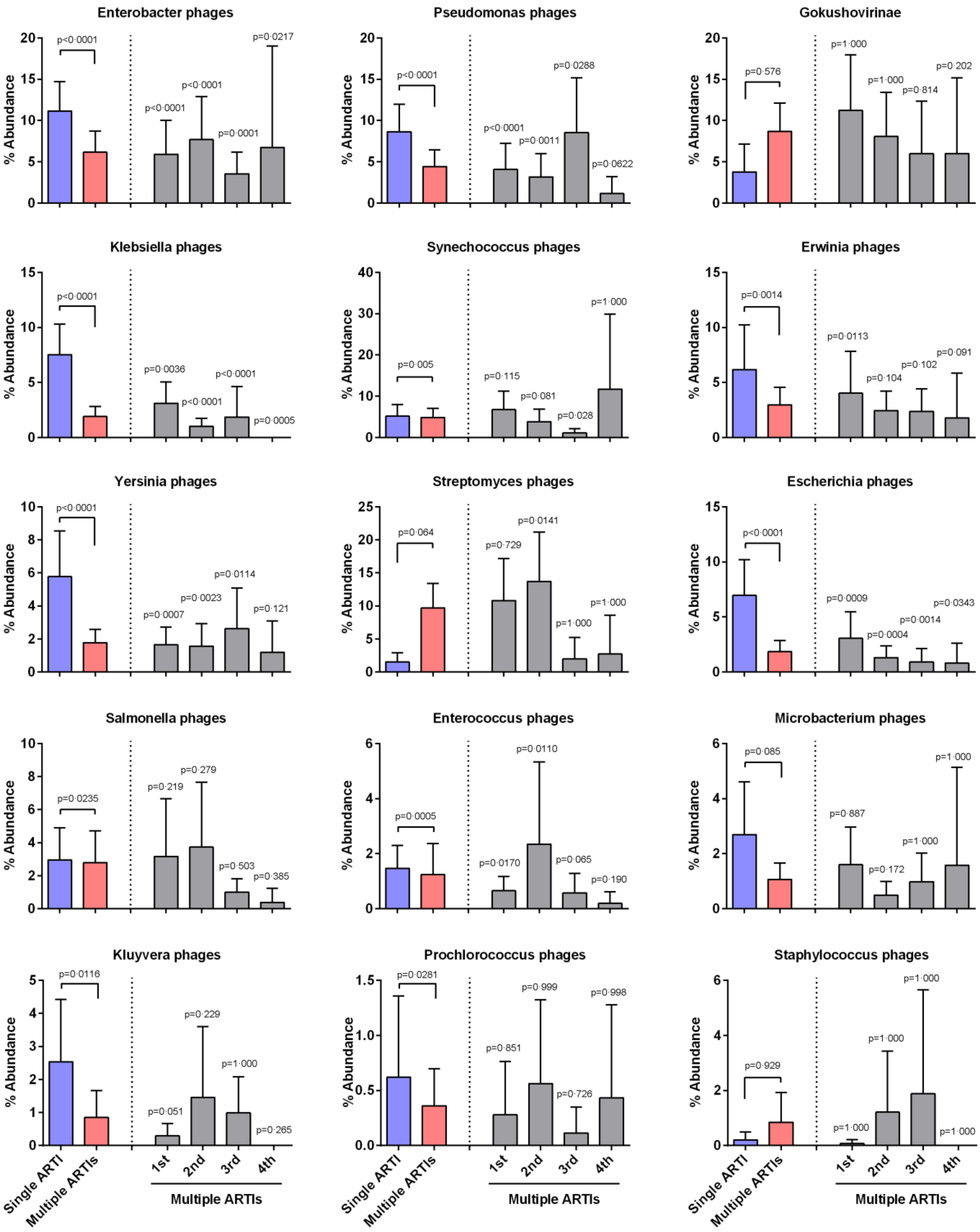

Supplementary Figure 3. Comparison of the abundance of different phages between multiple-ARTIs children and single-ARTI children. Phages with the top 15 prevalence were shown and analyses were performed using nonparametric Kruskal-Wallis Test corrected for multiple comparisons with Dunn's procedure, each column displays the mean abundance with the 95% CI. The number of patients in each group was the same with those in Figure 2.

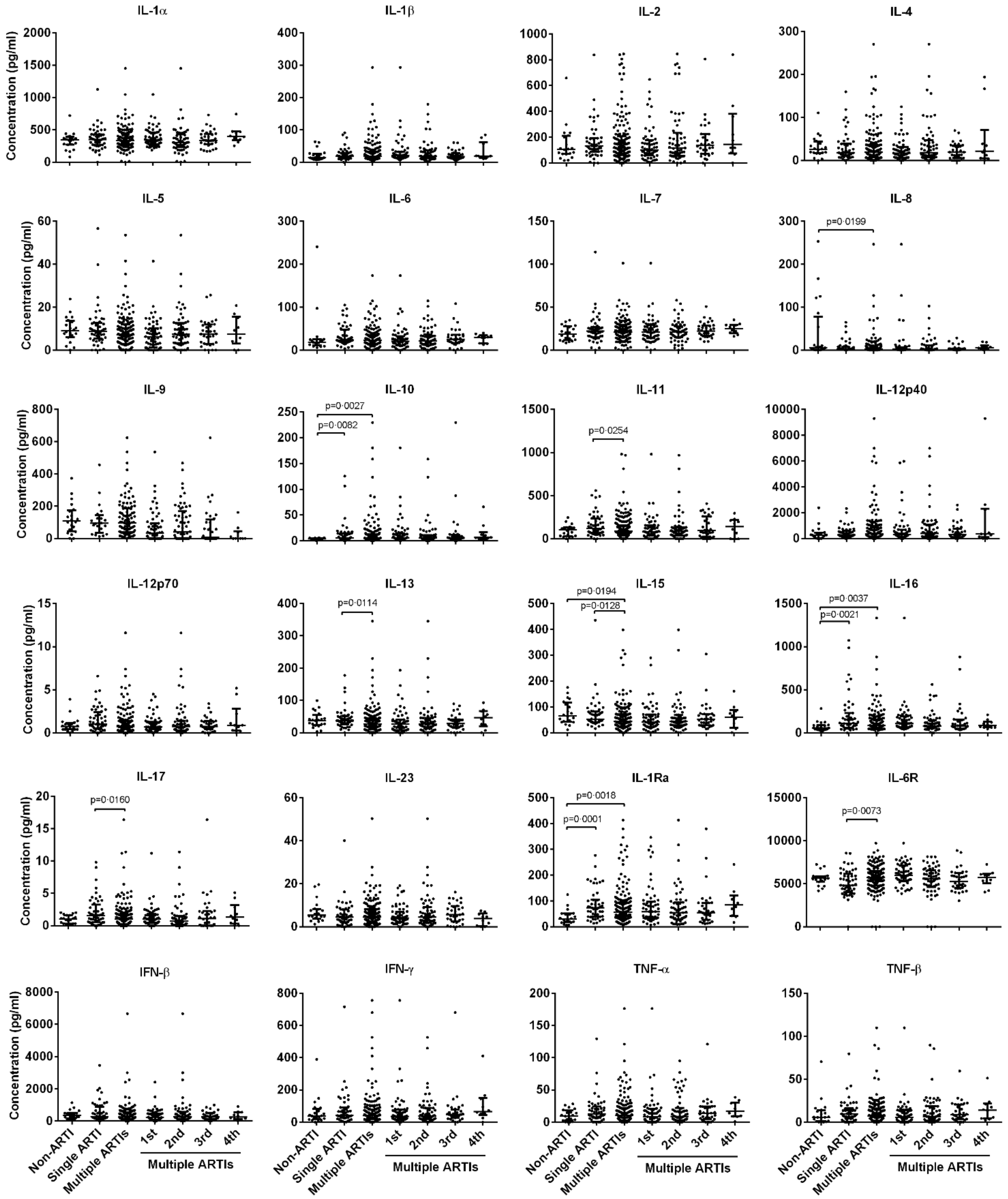

Supplementary Figure 4. Comparison of serum levels of 46 cytokines among children with single, multiple ARTIs, and healthy control. The serum levels of each cytokine at each episode of the multiple-ARTIs children were shown. The number of patients in each group is the same as those in Figure 4. Nonparametric Kruskal-Wallis Test corrected for multiple comparisons with Dunn's procedure was used. Error bars indicate the standard deviation.

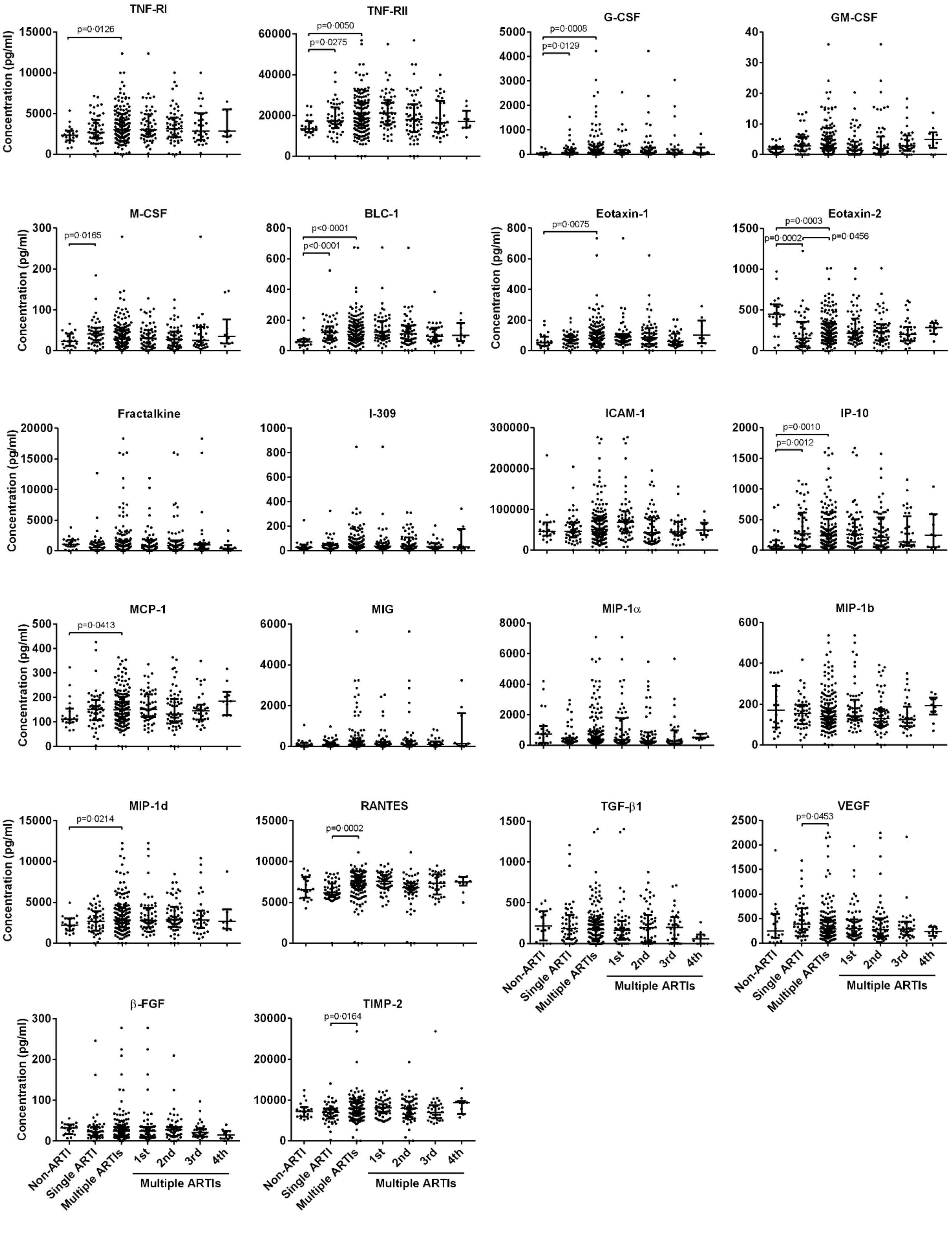

Supplementary Figure 4. (Continued)

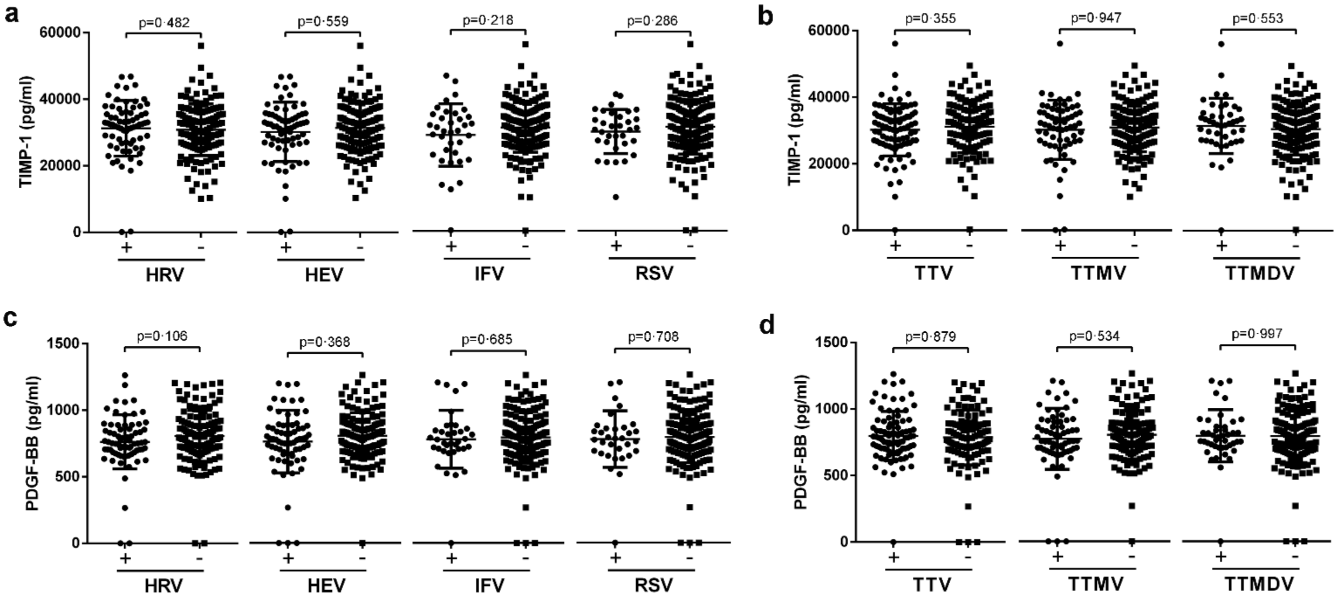

Supplementary Figure 5. Association of respiratory viruses with serum levels of TIMP-1 and PDGF-BB. Comparison of TIMP-1 and PDGF-BB serum levels between children who were positive (+) for four common respiratory viruses (i.e. HRV, HEV, IFV and RSV) (a and c), as well as three main anelloviruses (i.e. TTV, TTMV and TTMDV) (b and d), and those who were negative (-). Mann-Whitney U test was used. Error bars indicate the standard deviation.

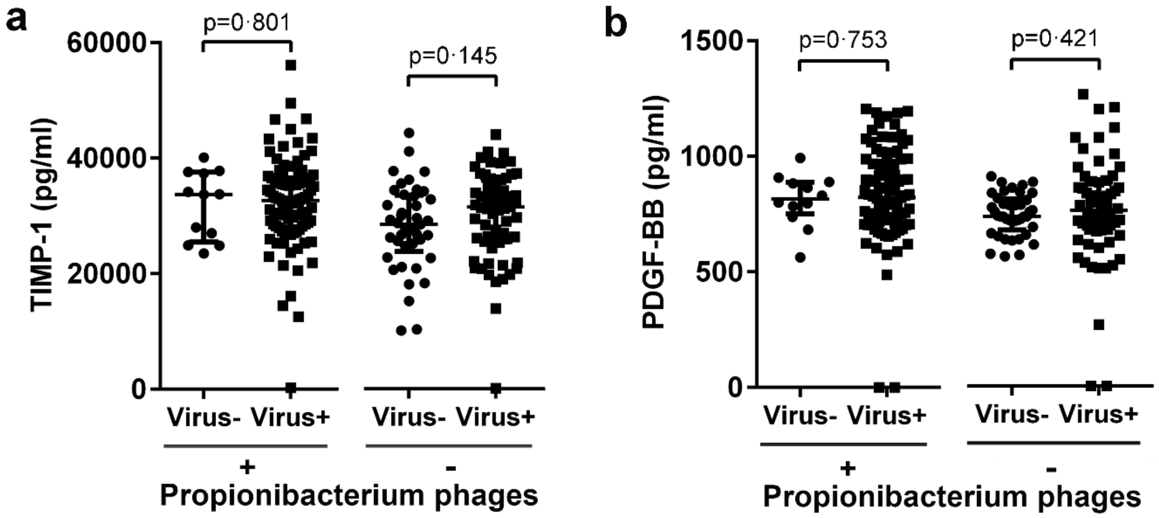

Supplementary Figure 6. Influence of other viral infections on the serum levels of TIMP-1 (a) and PDGF-BB (b) among children who were positive and negative for *Propionibacterium* phages. Samples were defined as virus positive (+) or virus negative (-) when the sample was positive or negative by NGS for any other eukaryotic viruses, respectively. Mann-Whitney U test was used. Error bars indicate the standard deviation.

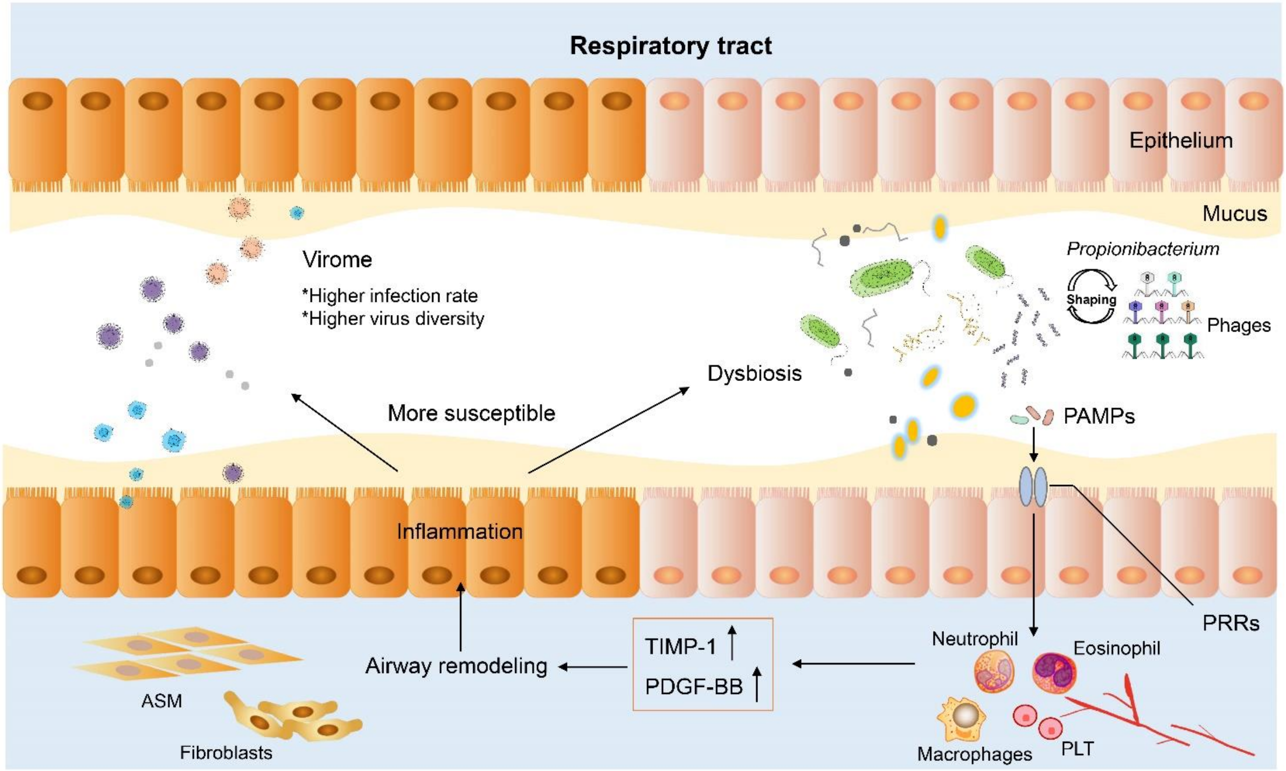

Supplementary Figure 7. Putative mechanism for the occurrence of multiple ARTIs. A balanced and stable resident microbial communities are crucial for resistance to infection and susceptibility of inflammation. Some bacteria are highly enriched when the microbial microbiota is unstable, allowing them to penetrate through the mucus layer. Then different pattern recognition receptors (PRRs) on the epithelial cells sense the microbiota through the pathogen-associated molecular pattern (PAMPs), and trigger the downstream immune responses from different cells. Of note, the significantly elevated TIMP-1 and PDGF-BB levels will lead to the airway remodeling by promoting the proliferation of smooth muscle cells (ASM), and increasing the airway fibroblast activity, and consequently render the airway susceptible for infections by common respiratory viruses or colonization by pathogenic bacteria.

References:

1. Wang, Y., *et al.* Metagenomic analysis of viral genetic diversity in respiratory samples from children with severe acute respiratory infection in China. *Clin Microbiol Infect* **22**, 458.e451-459 (2016).
2. Graf, E.H., *et al.* Unbiased Detection of Respiratory Viruses by Use of RNA Sequencing-Based Metagenomics: a Systematic Comparison to a Commercial PCR Panel. *J Clin Microbiol* **54**, 1000-1007 (2016).
3. Zhou, Y., *et al.* Metagenomics Study of Viral Pathogens in Undiagnosed Respiratory Specimens and Identification of Human Enteroviruses at a Thailand Hospital. *Am J Trop Med Hyg* **95**, 663-669 (2016).
4. Zoll, J., *et al.* Direct multiplexed whole genome sequencing of respiratory tract samples reveals full viral genomic information. *J Clin Virol* **66**, 6-11 (2015).
5. Thorburn, F., *et al.* The use of next generation sequencing in the diagnosis and typing of respiratory infections. *J Clin Virol* **69**, 96-100 (2015).
6. Wylie, K.M., *et al.* Metagenomic analysis of double-stranded DNA viruses in healthy adults. *BMC Biol* **12**, 71 (2014).
7. Lysholm, F., *et al.* Characterization of the viral microbiome in patients with severe lower respiratory tract infections, using metagenomic sequencing. *PLoS One* **7**, e30875 (2012).
8. Wylie, K.M., Mihindukulasuriya, K.A., Sodergren, E., Weinstock, G.M. & Storch, G.A. Sequence analysis of the human virome in febrile and afebrile children. *PLoS One* **7**, e27735 (2012).
9. Yang, J., *et al.* Unbiased parallel detection of viral pathogens in clinical samples by use of a metagenomic approach. *J Clin Microbiol* **49**, 3463-3469 (2011).
10. Willner, D., *et al.* Metagenomic analysis of respiratory tract DNA viral communities in cystic fibrosis and non-cystic fibrosis individuals. *PLoS One* **4**, e7370 (2009).

11. de Filippis, I., de Andrade, C.F., Caldeira, N., de Azevedo, A.C. & de Almeida, A.E. Comparison of PCR-based methods for the simultaneous detection of Neisseria meningitidis, Haemophilus influenzae, and Streptococcus pneumoniae in clinical samples. *Braz J Infect Dis* **20**, 335-341 (2016).
12. Arunrut, N., Kiatpathomchai, W. & Ananchaipattana, C. Multiplex PCR assay and lyophilization for detection of Salmonella spp., Staphylococcus aureus and Bacillus cereus in pork products. *Food Sci Biotechnol* **27**, 867-875 (2018).
13. Nam, Y.R.*, et al.* Degenerate PCR primer design for the specific identification of rhinovirus C. *J Virol Method* **214**, 15-24 (2015).
