# Supplementary material for: Altered respiratory virome and serum cytokine profile associated with recurrent respiratory tract infections in children
Source: Nat Commun. 2019 May 23;10:2288. doi: 10.1038/s41467-019-10294-x (PMC6533328; doi:10.1038/s41467-019-10294-x)
Supplement: Supplementary file 3 — Reporting Summary [file 41467_2019_10294_MOESM3_ESM.pdf]

nature research

Corresponding author(s): Chiyu Zhang & Jian_Hua Wang

Last updated by author(s): Apr 27, 2019

Reporting Summary

Nature Research wishes to improve the reproducibility of the work that we publish. This form provides structure for consistency and transparency in reporting. For further information on Nature Research policies, see Authors & Referees and the Editorial Policy Checklist.

Statistics

For all statistical analyses, confirm that the following items are present in the figure legend, table legend, main text, or Methods section.

n/a	Confirmed	
☐	☒	The exact sample size (*n*) for each experimental group/condition, given as a discrete number and unit of measurement
☐	☒	A statement on whether measurements were taken from distinct samples or whether the same sample was measured repeatedly
☐	☒	The statistical test(s) used AND whether they are one- or two-sided<br>*Only common tests should be described solely by name; describe more complex techniques in the Methods section.*
☐	☒	A description of all covariates tested
☐	☒	A description of any assumptions or corrections, such as tests of normality and adjustment for multiple comparisons
☐	☒	A full description of the statistical parameters including central tendency (e.g. means) or other basic estimates (e.g. regression coefficient) AND variation (e.g. standard deviation) or associated estimates of uncertainty (e.g. confidence intervals)
☐	☒	For null hypothesis testing, the test statistic (e.g. *F*, *t*, *r*) with confidence intervals, effect sizes, degrees of freedom and *P* value noted<br>*Give P values as exact values whenever suitable.*
☒	☐	For Bayesian analysis, information on the choice of priors and Markov chain Monte Carlo settings
☒	☐	For hierarchical and complex designs, identification of the appropriate level for tests and full reporting of outcomes
☒	☐	Estimates of effect sizes (e.g. Cohen's *d*, Pearson's *r*), indicating how they were calculated

Our web collection on statistics for biologists contains articles on many of the points above.

Software and code

Policy information about availability of computer code

Data collection	The clinical data were collected from the medical records of all patients. The NGS data were collected using the BGI-Seq500 platform (BGI, China). The expression data of cytokines were collected using Mepix (InnoScan 300 Microarray Scanner).
Data analysis	The raw NGS data was filtered by SOAPnuke software; Human host sequences were subtracted by SNAP software; and reads were assembled into contigs by Minimo9 and IDBA-UD. All statistical tests were performed using the SPSS19.0 or R package vegan 2.4-4; heat-map was obtained Origin Pro 2017, other graphs were generated by Origin Pro 2017 or GraphPad Prism 6; ROC curve and logistic regression analysis were obtained by SPSS19.0.

For manuscripts utilizing custom algorithms or software that are central to the research but not yet described in published literature, software must be made available to editors/reviewers. We strongly encourage code deposition in a community repository (e.g. GitHub). See the Nature Research guidelines for submitting code & software for further information.

Data

Policy information about availability of data

All manuscripts must include a data availability statement. This statement should provide the following information, where applicable:

- Accession codes, unique identifiers, or web links for publicly available datasets
- A list of figures that have associated raw data
- A description of any restrictions on data availability

All the sequencing data excluding human sequences were deposited in the CNSA (https://db.cngb.org/cnsa/) of CNGBdb (project number CNP0000429) under the accession number CNS0092588-CNS0092799. All the software used in this study are available from open source.

Field-specific reporting

Please select the one below that is the best fit for your research. If you are not sure, read the appropriate sections before making your selection.

☒ Life sciences ☐ Behavioural & social sciences ☐ Ecological, evolutionary & environmental sciences

For a reference copy of the document with all sections, see nature.com/documents/nr-reporting-summary-flat.pdf

Life sciences study design

All studies must disclose on these points even when the disclosure is negative.

Sample size

A total of 4407 children with ARTI were recruited in this study and 192 children were found to experience two or more ARTI episodes. Among them, 160 children had two ARTI episodes, 23 had three, 8 had four and one child had five. To investigate the respiratory virome associated with multiple ARTIs, all 32 other children experienced three to five episodes were selected for meteogenomic sequencing, together with 30 randomly selected children experienced two episodes for comparison. One child having two ARTIs was excluded from the final analysis due to failure in subsequent next generation sequencing (NGS), and thus results from a total of 61 multiple-ARTI children were presented. For comparison, 50 children who experienced only one ARTI episode during this study period (known as the single-ARTI group) and matched the multiple-ARTIs group with respect to age, gender, clinical presentations and laboratory tests were selected. Two of these were excluded from the final analysis due to failure in subsequent NGS, and thus results from a total number of 48 subjects were presented. For cytokine assay, a total of 212 ARTI serum samples (from the same individuals that used for respiratory virome study) including 48 from single ARTI children, 164 from multiple ARTIs children, and 20 healthy controls (non-ARTI group) were initially used for cytokine profile measurement. Five multiple-ARTI samples were excluded due to poor quality of cytokine assay, and thus results from 207 ARTI samples were presented.
The sample size in each group were comparable with each other and exceed all previously studies. The results clearly support the conclusions.

Data exclusions

One child having two ARTIs was excluded from the final analysis due to failure in subsequent next generation sequencing (NGS), and thus results from a total of 61 multiple-ARTI children were presented. Two of 50 children with one ARTI were excluded from the final analysis due to failure in subsequent NGS, and thus results from a total number of 48 subjects were presented. For the cytokine assay, five multiple-ARTI samples were excluded due to poor quality , and thus results from 207 ARTI samples were presented. No data were excluded from the subsequent analyses.

Replication

In this study, we characterized the respiratory virome and cytokine levels in children with multiple ARTIs. For metagenomic analyses and virome, we used a well-established method that has been widely used, it is not involved in the repeated experiments. For the determination of cytokine levels, we used a proteomic chip-based cytokine assay, and each immune mediator was measured in quadruplicates on the chips.

Randomization

We investigated the respiratory virome and immune profiles of single and multiple ARTIs among children from 2009 to 2015. The groups were allocated based on the times of ARTI episodes (from one to five times) during this study period. We included all involved children in the groups of three to five ARTIs. For the group of two ARTI episodes, 30 children were selected for compatibility with other groups (3 to 5 ARTI episodes). In addition, 50 children with one ARTI were randomly selected, and the number was matched the multiple-ARTIs group with respect to age, gender, clinical presentations and laboratory tests . Furthermore, 20 healthy children were included. We think our grouping was convincing.

Blinding

The aim of this study is to investigate the respiratory virome and immune profiles of single and multiple ARTIs among children, and characterize potential predictors for multiple ARTIs. We didn't know any information on the respiratory virome and immune profile of each sample before the NGS and cytokine assay; so blinding was not relevant to this study.

Reporting for specific materials, systems and methods

We require information from authors about some types of materials, experimental systems and methods used in many studies. Here, indicate whether each material, system or method listed is relevant to your study. If you are not sure if a list item applies to your research, read the appropriate section before selecting a response.

Materials & experimental systems

n/a	Involved in the study	
☒	☐	Antibodies
☒	☐	Eukaryotic cell lines
☒	☐	Palaeontology
☒	☐	Animals and other organisms
☐	☒	Human research participants
☒	☐	Clinical data

Methods

n/a	Involved in the study	
☒	☐	ChIP-seq
☒	☐	Flow cytometry
☒	☐	MRI-based neuroimaging

Human research participants

Policy information about studies involving human research participants

Population characteristics

A total of 4407children from 2009-2015 with ARTIs were used in this study, and finally 212 swab samples from 109 children were

Population characteristics	subjected to metagenomic analyses, 227 sera were subjected to proteomic chip-based cytokine assay. The detailed demographic information and clinical features of involved children were provided in the Table 1 and Figure 1.
Recruitment	The details of recruitment process were described in the methods and Figure 1 of the manuscript. In brief, we include 62 children who had more than one ARTI episodes (known as the multiple-ARTI group) from 2009-2015 , and for comparison, 50 children who experienced only one ARTI episode during this study period (known as the single-ARTI group) and matched the multiple-ARTIs group with respect to age, gender, clinical presentations and laboratory tests were selected.
Ethics oversight	This study was approved by the Ethics Committees of Shanghai Public Health Clinical Center and Shanghai Nanxiang Hospital

Note that full information on the approval of the study protocol must also be provided in the manuscript.
